# Choosing the Optimal Trigger Point for Analysis of Movements after Stroke Based on Magnetoencephalographic Recordings

**DOI:** 10.4061/2010/467673

**Published:** 2010-01-13

**Authors:** Guido Waldmann, Michael Schauer, Hartwig Woldag, Horst Hummelsheim

**Affiliations:** ^1^Neurologisches Rehabilitationszentrum Leipzig, University of Leipzig, Muldentalweg 1, 04828 Bennewitz, Germany; ^2^MediClin Reha-Zentrum Bad Düben, Gustav-Adolf-Straße 15, 04849 Bad Düben, Germany; ^3^Max Planck Institute for Human Cognitive and Brain Science, P.O. Box 500 355, 04303 Leipzig, Germany

## Abstract

The aim of this study was to select the optimal procedure for analysing motor fields (MF) and motor evoked fields (MEF) measured from brain injured patients. Behavioural pretests with patients have shown that most of them cannot stand measurements longer than 30 minutes and they also prefer to move the hand with rather short breaks between movements. Therefore, we were unable to measure the motor field (MF) optimally. Furthermore, we planned to use MEF to monitor cortical plasticity in a motor rehabilitation procedure. Classically, the MF analysis refers to rather long epochs around the movement onset (M-onset). We shortened the analysis epoch down to a range from 1000 milliseconds before until 500 milliseconds after M-onset to fulfil the needs of the patients. Additionally, we recorded the muscular activity (EMG) by surface electrodes on the extensor carpi ulnaris and flexor carpi ulnaris muscles. Magnetoencephalographic (MEG) data were recorded from 9 healthy subjects, who executed horizontally brisk extension and flexion in the right wrist. Significantly higher MF dipole strength was found in data based on EMG-onset than in M-onset based data. There was no difference in MEF I dipole strength between the two trigger latencies. In conclusion, we recommend averaging in respect to the EMG-onset for the analysis of both components MF as well as MEF.

## 1. Introduction

Improvement of hand function is important for regaining independency and social reintegration after stroke. Modern therapeutic approaches are based on the knowledge of motor learning and neuronal plasticity. To further increase the efficiency of therapeutic interventions, we have to learn more about the immediate effects of these interventions on the central motor system. The Magnetoencephalography (MEG) with its high spatial and temporal resolution is a usable tool for visualisation of cortical activity and its changes contemporary with functional improvements of the upper extremity in stroke patients in a rehabilitation context.

MEG is an important method for investigating the fast changing brain activity. From the experimental point of view, one separates spontaneous and event-related activity. The latter is evoked by certain stimulation whereas the first covers all the other activity. The onset of a presentation like a picture or sound can be defined on technical terms. The onset of a self-paced movement, however, has to be defined with physiological terms, in this case movement or electromyogram (EMG). One can record both measures simultaneously and use the onsets as triggers for averaging. The aim of this study was to investigate whether both measures serve this purpose equally well or not. The magnetic fields related to self-paced movements characteristically consist of the readiness field (RF), the motor field (MF), and a series of motor evoked fields (MEF). The readiness field starts 2000–500 milliseconds prior to EMG-onset [1]. The MF appears immediately prior to EMG-onset of the active muscle, and MEFs were observed after the EMG-onset. The MF is caused by activity in the region of the contralateral primary motor cortex [2–4]. The first motor evoked field (MEF I) after the EMG-onset is the strongest and most durable signal [4–6] and is thought to be generated mainly by proprioceptive input to the Brodmann area 3a [2, 6–8].

Investigations of MF and MEF I play a central role in the research of sensorimotor coupling and motor learning. Therefore, we assume that the strength of the MF and the MEF components might be suitable as quantitative markers for progress in a motor rehabilitation program with patients. The onset of brisk self-paced movements can easily be identified by the EMG-onset alone. However, the EMG activity is weak and shows low or no frequency discharges in movements with no outer resistance applied, in passive movements or in slow movements. For that reason, recording of the physical hand movement is required and narrows the temporal search range for increased EMG activity. 

For both components MF and MEF I, focal centres of activity can be assumed. Previous fMRI-studies (functional magnetic resonance imaging) describe mainly two cerebral regions, one contralateral and another ipsilateral region, as being active in motor tasks [9–11]. Thus, we model both components by equivalent current dipoles (ECD). The corresponding current dipole strength is dependent on the size of the activated region and on the degree of activation within this region. Therefore, we expect an increase in dipole strength during the rehabilitation program due to the motor training. In the beginning, however, activation strength might be tiny. Consequently, we have to seek for the most optimal trigger point to capture even small brain activity. 

Because of the tight efferent coupling between neuronal activity in the primary motor cortex and the corresponding muscle activity, we hypothesize that computing the MF with respect to the EMG-onset shall lead to a larger, better synchronized effect compared to averages with respect to the movement-onset (M-onset, start of wrist motion). 

MEF I is thought to be generated by proprioceptive input in Brodmann's area 3a mainly by afferences from group I muscle spindles [12, 13]. Since muscle spindles are very sensitive to stretch, and respond even to displacements of a few micrometers, they respond to both the EMG activity and the real movement of the entire limb. Therefore, we assume that MEF I effects may not vary in dependence of the reference point used for averaging.

## 2. Materials and Methods

### 2.1. Subjects and Tasks

Magnetoencephalographic data were recorded from 9 healthy right handed subjects, aged 23 to 32 years, mean: 27 years. They had neither actual symptoms nor history of neurological disorders. Each person had to execute the movements in two sessions. Because of artefacts only 15 sessions could be included in the investigation. Written informed consent of the subjects and approval of the ethical committee are present.

**Figure 1:**
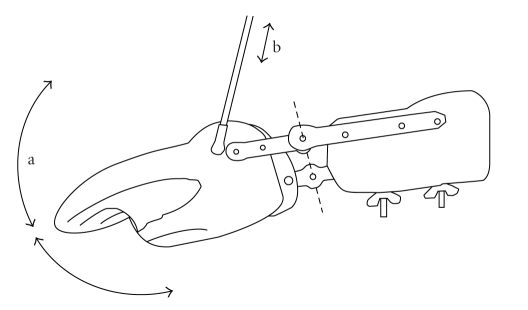
Scheme of the splint (top view) with a joint at the wrist. The right hand was positioned with the thumb upwards. The hand movement angle (a) is mechanically transmitted by the connecting rod (b) to a digital goniometric sensor over a distance of about 100 cm to reduce ferromagnetic artefacts.

Subjects were positioned in a comfortable seat stabilized by a vacuum mattress inside a magnetically shielded room (Vakuumschmelze GmbH, Hanau, Germany). The eyes were opened and the right distal arm and hand were fixed by a splint with a joint at the wrist, so that the hand movements were still possible but restricted to dorsal extensions and palmar flexions (Figure 1). This type of movement was chosen to allow future measurements with weak patients minimizing the gravitational influence to their hand. The hand was positioned with the thumb upwards, so that the hand moved in the horizontal plain. The hand position was mechanically transmitted to an electronic goniometric sensor. In this way the wrist angle could be recorded together with the MEG data at the same sampling rate of 508.63 Hz. The resolution of the goniometry was 1.4 degrees per bit.

**Figure 2:**
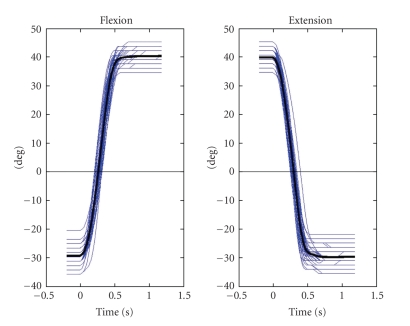
Time courses of the angular movements executed by one subject. The displayed traces are synchronized in time according to the procedure described in Section 2. Therefore, by definition, movement started at time zero. The thicker black line indicates the mean.

Subjects were instructed to carry out alternating, brisk and self-paced extensions and flexions of the right wrist. Before each movement, the wrist rested in an end position (flexion or extension) and then moved to the opposite extension or flexion position (Figure 2). Between the movements a resting period of about 5 seconds was requested. The movement sequence was observed from outside the shielded room and commented occasionally to maintain the acceleration and resting period. Each session contains four blocks lasting 15 minutes each.

### 2.2. Data Acquisition

The MEG was continuously recorded using a 148-channel Magnes WHS 2500 whole head system (4D-NeuroImaging, San Diego, U.S.A). Data were sampled at rate of 508.63 Hz and recorded using a bandpass filter from 0.1 Hz to 100 Hz. Horizontal and vertical electrooculograms (EOG) were registered with two bipolar channels. Four bipolar surface EMGs were recorded (left and right extensor carpi ulnaris and flexor carpi ulnaris muscles) to exclude data with contralateral muscle activity. The corresponding Ag/AgCl-electrodes were mounted over the muscle bellies with a distance of 2 cm.

The head surface of each subject was digitized before measurement outside the MEG chamber using a 3D-Digitizer Fasttrak (Polhemus, Colchester, U.S.A). Subject's head position was measured before and after each experimental block by the head position indicator system of the MEG device. Blocks were excluded from further analysis when head positions varied by more than 5 mm.

### 2.3. Data Processing

The MEG-data were filtered off line with a 2–15 Hz bandpass filter (3rd-order Butterworth). To reduce artefacts by eye-blinks and movements a sliding standard deviation was estimated for both EOG-channels using a 200-millisecond time interval. Whenever a standard deviation signal exceeded a threshold of 40 *μ*V, all MEG-data of the corresponding interval were rejected. The influences of other magnetic artefacts (e.g., muscular activity of the neck and strong environmental magnetic fields changes) were reduced by applying the same method to all MEG-sensors using a threshold of 2000 fT in a 2000-millisecond time interval. Furthermore, another artefact rejection was applied to detect non functional MEG channels automatically. The procedure is based on correlation coefficients between vicinal sensors. Vicinal sensors are a group of 4–7 sensors, which share a conjoint sensor within a distance smaller than 40 mm. Such a sensor group picks up similar signals because they are all close to the same brain region. Therefore, recordings of such a group should be strongly correlated. If the median of correlations from the middle sensor of a group to all its neighbours falls below 0.75, the channel is considered to be non-functional and excluded from the further processing. Averaged epochs with a signal-to-noise ratio of less than two were excluded from the final localizing step.

Two trigger points were determined (a) the M-onset was estimated on the basis of the goniometric device data and (b) the EMG-onset was defined on the basis of the EMG recordings of the right extensor carpi ulnaris and flexor carpi ulnaris muscles. An M-onset was detected if the wrist angle varied by more than 1.4 degrees from the wrist position after a resting period of at least 2 seconds and if the angular speed exceeded 75 degrees/s. The direction of the movement was classified as extension or flexion, respectively. The determination of the EMG-onset included the following four stages: the EMG signal was (1) high pass filtered with a cutoff of 40 Hz, (2) rectified, (3) low pass filtered with the cutoff of 20 Hz, and (4) thresholded. The threshold was calculated as the mean EMG-activity within a 400-millisecond interval starting 200-millisecond before the movement onset. This procedure is adopted from Barrett et al. [14]. 

Finally, the MEG data have been averaged separately for each subject in four conditions: movement direction (flexion and extension) and trigger point (M-onset and EMG-onset). All epochs were 1400 milliseconds in length and started 1000 milliseconds before the corresponding trigger. Such short epochs were chosen to minimize the impact of artefacts, especially when investigating patients with movement disorders. In further pilot studies with patients suffering from central movement disorders, we observed increased variance in post movement time and shorter resting periods of about 2 seconds. Furthermore, even the healthy volunteers in this study tended to shorten the post movement resting time. Additionally, a 2 Hz-highpass filter was applied to the data to substitute the classical baseline correction [5], which could not be applied due to the short resting time between movements. The highpass filter leads to a reduction in amplitude of about 10% for each of both effects, the MF and the MEF I. 

The following procedure was conducted to all four conditions.

The exploration of movement related fields was performed in an analysis interval of −350 to +150 milliseconds around the trigger point. As a starting point we decided to model the measured field distribution by a pair of equivalent current dipoles. Both dipoles were initially placed symmetrically one per hemisphere to explain both contra- and ipsilateral activities. Dipolar activity was modelled in subsequent 20-millisecond-intervals. For each interval, position and orientation of the two dipoles were calculated. The forward model was based on a spherical volume conductor, which was fitted in radius and centre to the subject's individual head shape. This volume conductor provides sufficient accuracy as recently published by Scheler et al. [15]. The local maxima of the contralateral dipole magnitudes were estimated and their latencies identified as the latencies of the movement related fields (MF or MEF I). A similar technique was used in the analysis of passive wrist movements by Lange et al. [16]. The dipoles analysis was conducted using ASA software (A.N.T. Software B.V., Enschede, The Netherlands). 

Individual dipole locations of the four conditions (flexion/extension in M-onset as well as EMG-onset) were transformed into Montreal Standard Brain (MNI) coordinates [17] and compared between subjects. The difference in the median values of localization coordinates between extension and flexion directions was not great enough to exclude the possibility that the difference is due to random sampling variability (tested with Mann-Whitney Rank Sum Test). There is not a statistically significant difference for MF *P* = .79 and for MEF I *P* = .43 in EMG-onset (extension versus flexion) and no statistically significant difference for MF *P* = 1.0 and for MEF I *P* = .57 in M-onset (extension versus flexion).

There are also no significant differences in dipole strength (MF: EMG-onset extension versus flexion *P* = .92; MF: M-onset extension versus flexion *P* = .58; MEF I: EMG-onset extension versus flexion *P* = .31; MEF I: M-onset extension versus flexion *P* = .47). 

Therefore, extension and flexion movements had not to be calculated in a different matter. 

Macroanatomical labels (e.g., Brodmann's areas) were used to relate the activation to cortical sulci or deep brain nuclei. As the main use of labels is to identify activation as belonging to a functional area, macroanatomical labels are of most use when there is an established relationship between anatomy and function. This is usually the case for the deep brain nuclei, but the relationship between function and sulcal anatomy is much less clear. Nevertheless, it is reasonable to identify motor cortex activation according to its position in relation to the central sulcus.

The same applies to primary auditory cortex, which has a clear relationship with Heschl's gyrus, and to primary visual cortex, which can be identified by the position of the calcarine sulcus. These sulci and gyri are relatively invariant in position and configuration between individuals. However, most sulci in the brain are highly variable between subjects and even between hemispheres in a single subject [17]. The used forward model was based on a spherical volume conductor, which was fitted in radius and centre to the subject's individual head shape. This volume conductor provides sufficient accuracy as recently published by Scheler et al. [15]. For the transformation, individual spherical volume conductor has been transformed into equal size.

We decided to use the dipole model as a spatial filter to project the measured magnetic field onto a single current dipole placed within the contralateral motor area.

This approach is similar to the source space projection method by Scheler et al. [15] with the difference that the signal space is derived from a prior knowledge on the source position rather than from the signal itself. Note that the spatial resolution of this method is limited. The separation limit is about 2-3 cm [18]. 

For this purpose the dipoles locations were taken from previous publications [2, 19]. The MF is assumed to originate from (*x*_NE_, *y*_NE_, *z*_NE_) = (17, 30, 93 mm) and the MEF I from (*x*_NE_, *y*_NE_, *z*_NE_) = (3, 19, 85 mm) with respect to the MNI brain. 

The statistical analysis of dipole-magnitudes (nAm) of MF or MEF I between M-onset and EMG-onset was carried out with the Wilcoxon rank test for paired data. The level of significance was set to *P* = .05.

## 3. Results and Discussion

The analysis was focused on the comparison of MF and MEF I magnitudes depending on the trigger type at M-onset or EMG-onset. An especial challenge and also limitation of the investigation is a regular wrist movement.

Figure 2 displays the set of time courses recorded from the goniometer for the typical subject. The picture demonstrates two aspects: first, our definition of the movement onset results in a reasonable time alignment of epochs and second, the movements were executed in a rather stereotypical manner. 

**Figure 3:**
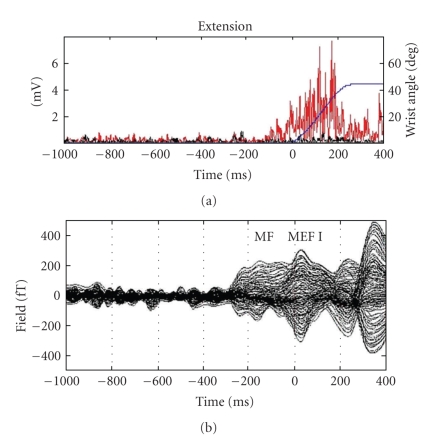
Mean wrist extensions of a typical subject: (a) Time course of electromyography signals in extensor and flexor muscles and wrist angle (one exemplary epoch; red: EMG signal of the extensor carpi ulnaris muscle; black: EMG signal of the flexor carpi ulnaris muscle; blue: wrist angle). (b) Overlay of magnetoencephalographic signals (148 channels) averaged with respect to movement onset. Time 0 indicates the extension M-onset.

Figure 3(a) displays the mean time courses of the goniometer (blue line) and both ipsilateral EMG channels for a typical subject and the extension movement. The flexor EMG is displayed in black and the extensor EMG in red. Figure 3(b) shows an overlay of the mean time courses of all magnetic channels averaged with respect to the M-onset.

The mean values across subjects of the dipole magnitudes for both effects, MF and MEF I, and both average trigger points, EMG-onset and M-onset, are shown in Table 1. The MF dipole magnitudes averaged in respect to the EMG-onset are significantly larger than when averaged with respect to M-onset, *P* < .05 (.008). The MEF I dipole magnitudes did not differ significantly between both trigger points (Table 1).

The comparison of MF and MEF I magnitudes depending on the trigger type at M-onset or EMG-onset confirms our hypotheses: the MF dipole magnitude determined in the EMG-onset averaging is indeed significantly larger (10.7 ± 10.0 nAm) than those in the M-onset averaging (5.6 ± 3.6 nAm). The MEF I dipole magnitudes, however, do not depend on trigger point. 

### 3.1. MF

The dipoles were of a larger magnitude in the EMG-onset averaging compared to the M-onset averaging. This finding points to a tight coupling between motor cortex activity and neuromuscular transmission and corresponds nicely to the data of Cheyne and Weinberg [2]. They reported a dipole magnitude of 9.5 ± 2.6 nAm. Additionally, the motor field latency data from our study (Table 2) matches the results of previous investigations [4, 5, 20–24], although they refer to other types of movement, either tapping or elbow flexion. The reported MF latencies in M-onset averaging were about 80 to 250 milliseconds and in EMG-onset averaging about 120 to 160 milliseconds. The smaller variation for the latter latencies does also support the view that the EMG-onset represents the superior trigger point for computing the MF effect.

### 3.2. MEF I

The dipole magnitudes did not depend on the trigger point. It is generally agreed that the afferent cortical activity is tightly coupled to muscle spindle signals, the most likely source of the afferent information about fascicle activity. Muscle spindles are very sensitive to stretch and respond even to displacements of few micrometers [25–27]. Our values of MEF I dipole magnitude correspond to the values of Cheyne and Weinberg [2], who stated 20.9 ± 2.5 nAm with the latency of 90–130 milliseconds after EMG-onset. The difference between the MEF I dipole magnitude and the MF dipole magnitude replicated previous results nicely. The measured MEF I latencies correspond closely to former investigations [2, 4, 6, 20, 24]. 

The delay between EMG-onset and M-onset corresponds to the myo-tendinous and myo-fascial force transmission, which leads to a time delay between neuromuscular transmission, myofiber calcium influx, muscle contraction, and the resulting movement which adds up to values between 30 milliseconds and 100 milliseconds [28, 29]. The weak wrist movements without greater force production and without high frequency of myoelectric discharges in our study may cause a certain intra- and interindividual variability of the time delay between EMG-onset and M-onset. This explanation is supported by the debate about the relation between produced force and EMG magnitude. The proprioceptive feedback starts simultaneously to or immediately after EMG-onset because of the responsiveness of muscle spindles and produces a first motor evoked field in the sensory cortical fields/ Brodmann area 3a. 

For the analysis of motor field (MF) evoked by self-paced wrist movements, averaging with respect to the EMG-onset is superior to the alternative based on movement onsets. The analysis of motor evoked field (MEF I) does not depend on the choice of the average trigger point.

## Figures and Tables

**Table 1:** Mean dipole magnitudes across the subject's motor fields (MF) and Motor evoked fields I (MEF I) in movement onset (M-onset) and EMG-onset per wrist flexion and extension (mean ± standard deviation).

	Dipole-Magnitudes [nAm]	
	M-onset	EMG-onset
MF	5.6 ± 3.6	10.7 ± 10.0
MEF I	28.7 ± 17.7	27.4 ± 15.0

**Table 2:** Mean latencies across the subject's motor fields (MF) and Motor evoked fields I (MEF I) in movement onset (M-onset) and EMG-onset per wrist flexion and extension (mean ± standard deviation).

	Latencies [ms]	
	M-onset	EMG-onset
MF	−222 ± 74	−112 ± 88
MEF I	−26 ± 31	60 ± 28
